# Polyphenols from *Erythrina crista-galli*: Structures, Molecular Docking and Phytoestrogenic Activity

**DOI:** 10.3390/molecules21060726

**Published:** 2016-06-03

**Authors:** Naglaa S. Ashmawy, Mohamed L. Ashour, Michael Wink, Mohamed El-Shazly, Fang-Rong Chang, Noha Swilam, Ashraf B. Abdel-Naim, Nahla Ayoub

**Affiliations:** 1 Department of Pharmacognosy, Faculty of Pharmacy, Ain Shams University, Cairo 11566, Egypt; naglaa.saad@pharma.asu.edu.eg (N.S.A.); ashour@pharma.asu.edu.eg (M.L.A.); elshazly7@yahoo.com (M.E.-S.); 2 Department of Biology, Institute of Pharmacy and Molecular Biotechnology, Heidelberg University, Heidelberg 69120, Germany; wink@uni-heidelberg.de; 3 Graduate Institute of Natural Products, Kaohsiung Medical University, Kaohsiung 807, Taiwan; aaronfrc@kmu.edu.tw; 4 Department of Pharmacognosy, Faculty of Pharmacy, British University of Egypt (BUE), Cairo 11837, Egypt; Noha.Swilam@bue.edu.eg; 5 Department of Pharmacology and Toxicology, Faculty of Pharmacy, Ain Shams University, Cairo 11566, Egypt; abnaim@pharma.asu.edu.eg or abnaim@yahoo.com; 6 Department of Pharmacology, Faculty of Medicine, Umm Al Qura University, Makkah 21955, Saudi Arabia

**Keywords:** *Erythrina crista-galli*, polyphenols, molecular docking study, phytoestrogenic activity

## Abstract

Objectives: The current study aimed at exploring the secondary metabolites content of *Erythrina crista-galli* aqueous methanol extract and assessing its phytoestrogenic and cytoprotective activities. Methods: Isolation of the compounds was carried out using conventional chromatographic techniques. The structures of the isolated compounds were elucidated based on the UV, NMR spectral data along with their mass-spectrometric analyses. The phytoestrogenic activity was evaluated *in-silico* and *in vitro* using the *Arabidopsis thaliana* pER8: GUS reporter assay and the proliferation-enhancing activity of MCF-7 cells. Key findings: Phytochemical investigation of *E. crista-galli* aqueous methanol extract resulted in the isolation and identification of five flavonoids. The plant extract and its fractions showed significant estrogenic activities compared to controls. Conclusion: Five flavonoids were identified from *E. crista-galli* aqueous methanol extract. To the best of our knowledge, among these flavonoids, apigenin-7-*O*-rhamnosyl-6-*C*-glucoside was isolated for the first time from nature. Moreover, luteolin-6-*C*-glucoside was isolated for the first time from this plant. The plant revealed promising phytoestrogenic activities. This gives rationale to some of its pharmacological properties and suggests additional phytoestrogenic effects, which have not been reported yet.

## 1. Introduction

Phenolic compounds are the most widely distributed secondary metabolites in terrestrial plants. They are typical multi-target compounds exerting multiple biological effects [1]. These effects, such as antibacterial [2], antioxidant [3], phytoestrogenic [4,5], cytotoxic [6] and hepatoprotective activities, have been thoroughly investigated [7,8].

The family Fabaceae (the legume family) includes about 730 genera with more than 19,500 species. Plants belonging to this family biosynthesize and accumulate large amounts of phenolics, particularly flavonoids, phenolic acids and tannins [9]. Red clover (*Trifolium pratense*), rich in phytoestrogenic isoflavones, such as genistein and daidzein, and soybean (*Glycine max*), with the methylated isoflavones, biochanin A and formononetin [10], are known examples of phenolic-rich and bioactive legumes. The heart wood of *Acacia* [11] and the roots of *Glycyrrhiza glabra* are important sources of phenolic compounds [12]. Some of these phenolics exhibit a potent hepatoprotective effect [13].

The genus *Erythrina* (Fabaceae) contains more than 100 species distributed in the tropics and subtropics of America, Africa and Australasia [14]. The origin of the name *Erythrina* comes from the Greek word “erythros” which means red, referring to the bright red flowers of the genus trees. Many *Erythrina* species, with their attractive flowers, are cultivated as ornamental plants in many tropical and subtropical areas of the world. These species have been widely used in traditional medicine for the treatment of insomnia, malaria fever, venereal disease, asthma and toothache [15]. Most of the phytochemical investigations on *Erythrina* species were directed towards their neurotoxic alkaloids, which are a special class of isoquinoline alkaloids [16,17,18]. Additionally, some *Erythrina* species were reported to contain a novel class of phytoestrogenic alkaloids, named as erythroidine alkaloids [19]. Furthermore, studies on *E. poeppigiana* indicated the presence of phenolic constituents with inhibitory potential against human glyoxalase I [20]. Other secondary metabolites such as flavonoids [21], isoflavonoids [22,23], pterocarpanes [24,25], flavanones [26,27], isoflavans [28], chalcones [29,30] and cinnamoyl phenols [31,32] were also reported.

*Erythrina* extracts exhibit a wide range of pharmacological properties, including antibacterial [22,33], antimalarial [34], antioxidant [17,35], anti-inflammatory [36], cytotoxic [37,38,39], tyrosine-protein phosphatase inhibitory [40], phytoestrogenic [19,41,42], anti-osteoporotic [43] and neurotoxic activities [44]. *E. crista-galli* is widely distributed in subtropical and tropical regions of South America. It is known as Cockspur Coral Tree and commonly called “Corticeira” in Brazil. Its bark is used for the treatment of many ailments related to rheumatism, and for hepatitis [45]. Phytochemical studies on the underground parts revealed several *Erythrina* alkaloids, isoflavonoids and pterocarpans [46]. The present study focused on the identification of the major phenolic secondary metabolites from the aerial parts (leaves) of *Erythrina crista-galli*. Potential phytoestrogenic activities of *E. crista-galli* leaves extracts and fractions were also investigated.

## 2. Results and Discussion

Apigenin-7-*O*-rhamnosyl-6-*C*-glucoside (**1**), a minor compound, was obtained as an amorphous yellow powder, which appeared as a dark purple spot on the PC and turned yellow upon exposure to ammonia vapors, under UV light. R_f_ = 0.34 (6% AcOH) and 0.27 (BAW). The UV spectral data of the compound revealed two distinct maxima, one at 266 nm for band II and one at 336 nm for band I, indicating a flavone skeleton. A bathochromic shift (50 nm) was observed in band I upon addition of sodium methoxide, indicating the probable presence of a free hydroxyl group at position 4′. No bathochromic shift was observed in band I on the addition of aluminium chloride, indicating the absence of *ortho*-dihydroxyl groups. The absence of *ortho*-dihydroxyl groups was further confirmed by the absence of the bathochromic shift in band I in the sodium acetate/boric acid spectrum. No bathochromic shift was observed in band II in the presence of sodium acetate, indicating the absence of a free hydroxyl group at position 7. Negative ESI-mass spectral analysis ([M − 1] at *m*/*z* = 577, corresponding to a molecular mass of 578), together with the above given analytical data, suggested an apigenin molecule containing a hexoside and rhamnoside moieties; at least one of these moieties occupies position 7.

The proposed structure of this minor constituent was then proved by ^1^H-NMR and COSY analysis (DMSO-*d*_6_, room temperature). The presence of 7-*O*-substitution of apigenin was concluded from the present downfield shifts of the aromatic protons on ring A, which showed a signal at δ 6.72 (1H, s, H-8). The identity of apigenin was confirmed by the presence of signals at δ 6.91 (2H, d, *J* = 9 Hz, H-3′ and H-5′), 7.92 (2H, d, *J* = 9 Hz, H-2′ and H-6′) and 6.91 (1H, s, H-3). The absence of the H-6 signal indicated *C*-substitution at the position of this carbon. Moreover, the anomeric proton of the hexosyl moiety displayed a signal at δ 4.57 (1H, d, *J* = 9 Hz, Hglu-1′′). The appearance of the H-1′′ proton of this moiety at δ 4.57 suggests a *C*-hexosyl linkage, because in case of *O*-glycosidic linkage, the same proton should appear at a downfield location. That the hexoosyl moiety in compound **1** exists in β-configuration, was evidenced by the splitting pattern of its anomeric proton resonance into doublet at δ 4.57 (1H, d, *J* = 7 Hz, H-1′′), which indicated an axial-axial coupling between H-1′′ and H-2′′ sugar protons. The anomeric proton of rhamnose (identified as rhamnose from the methyl substituent) displayed a signal at δ 5.47 (1H, d, *J* = 2 Hz, Hrha-1′′′), a location which is downfield enough to suggest an *O*-type of glycosidation. A *C*-rhamnosyl substitution will bring that anomeric proton comparatively upfield (e.g., at δ = 4.8 ppm). Other signals of sugar protons appeared between 3.1–4.2 and 1.2 (3H, d, *J* = 6.0 Hz, CH_3_-6′′′). Finally, enzymatic hydrolysis of compound **1** by α rhamnase enzyme yielded compound **2**.

**Figure 1:**
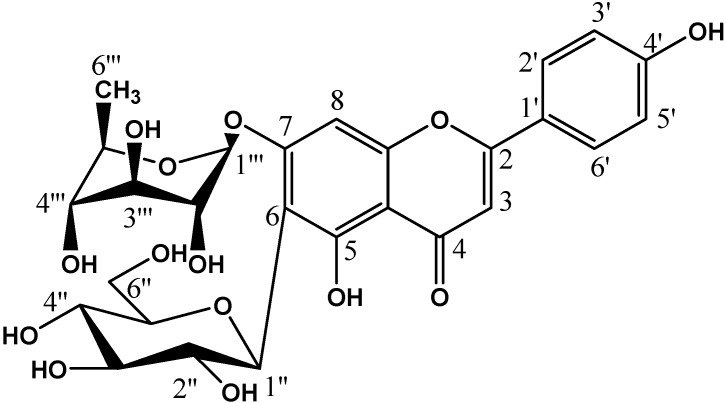
Chemical structures of the isolated compounds from *Erythrina crista-galli*. Compound **1**: apigenin-7-*O*-rhamnosyl-6-*C*-glucoside.

The above given data suggested, therefore, an apigenin-7-*O*-rhamnosyl-6-*C*-glucoside structure for the minor constituent (**1**), a new natural flavone glycoside (Figure 1) [47].

*Apigenin-6-C-glucoside* (**2**) (Figure 1) was obtained as an amorphous yellow powder, which appeared as a dark purple spot on the PC, which turned yellow upon exposure to ammonia vapors, as seen under UV light. R_f_ = 29 (6% AcOH) and 48 (BAW). ^1^H-NMR (500 MHz, DMSO-*d*_6_, room temp.) δ: 7.80 (2H, d, *J* = 8.5 Hz, 2′, 6′-H), 6.89 (2H, d, *J* = 8.4 Hz, 3′, 5′-H), 6.53 (1H, s, 3-H), 6.18 (1H, s, 8-H), 4.55 (1H, d, *J* = 9.8 Hz, 1′′-H), 4.03–3.11 (6H, m, sugar). ^13^C-NMR (500 MHz, DMSO-*d*_6_) δ: 163.32 (C-2), 102.60 (C-3), 181.73 (C-4), 160.64 (C-5), 109.77 (C-6), 163.32 (C-7), 95 (C-8), 157.38 (C-9), 102.95 (C-10), 121.46 (C-1′), 128.35 (C-2′, 6′), 116.53 (C-3′, 5′), 161.32 (C-4′), 81.41 (C-1′′), 70.44 (C-2′′), 74.25 (C-3′′), 70.20 (C-4′′), 79.54 (C-5′′), 61.37 (C-6′′). The UV (neat and after addition of shifting reagents) and NMR spectral data were identical to those reported for apigenin-6-*C*-glucoside (isovitexin) [48].

*Luteolin-6-C-glucoside* (**3**) (Figure 1) was obtained as an amorphous yellow powder, which appeared as a dark purple spot on the PC under UV light and turned yellowish green upon exposure to ammonia vapors and then turned dirty green after spraying with 1% methanol ferric chloride solution. R_f_ = 37 (6% AcOH) and 44 (BAW). ^1^H-NMR (500 MHz, DMSO-*d*_6_) δ: 7.38 (1H, dd, *J* = 2.5 Hz, 9.0 Hz, 6′-H), 7.38 (1H, d, *J* = 2.5 Hz, 2′-H), 6.89 (1H, d, *J* = 9.0 Hz, 5′-H), 6.52 (1H, S, 3-H), 4.45 (1H, d, *J* = 10.0 Hz, 1′′-H). ^13^C-NMR (500 MHz, DMSO-*d*_6_) δ: 163.45 (C-2), 103.75 (C-3), 182.54 (C-4), 160.57 (C-5), 107.72 (C-6), 164.79 (C-7), 93.76 (C-8), 157.24 (C-9), 102.46 (C-10), 121.56 (C-1′), 112.92 (C-2′), 145.50 (C-3′), 149.50 (C-4′), 115.35 (C-5′), 118.88 (C-6′), 81.18 (C-1′′), 70.36 (C-2′′), 73.86 (C-3′′), 71.16 (C-4′′), 78.69 (C-5′′), 61.44 (C-6′′).The UV (neat and after addition of shifting reagents) and NMR spectral data were similar to those reported for luteolin-6-*C*-glucoside (Isoorientin) [49], which is reported here for the first time in *Erythrina crista-galli* [48].

Compound **4** *5,7,4′-trihydroxyflavone* (apigenin) (Figure 1), was obtained as a yellow crystalline material, R_f_ = 49 (6% acetic acid) and 86 (BAW). Compound **4** appeared as a dark purple spot on the PC under UV. The ^1^H-NMR spectral data showed the following resonance peaks at δ (ppm) 6.03 (d, *J* = 2.5 Hz, H-6), 6.31 (d, *J* = 2.5 Hz, H-8), 6.64 (s, H-3), 6.88 (d, *J* = 8 Hz, H-3′, 5′), 7.85 (d, *J* = 8 Hz, H-2′, 6′). Comparison of the obtained spectral data of compound **4** with those reported for apigenin confirmed its identity [49]. Apigenin is reported here for the first time from *Erythrina crista-galli.*

Compound **5** *5,7,3′,4′-tetrahydroxyflavone* (luteolin) (Figure 1), was obtained as a yellow crystalline material, R_f_ = 66 (6% acetic acid) and 78 (BAW). Compound **5** appeared as a dark purple spot on the PC under UV. The ^1^H-NMR spectral data showed the following resonance peaks at δ (ppm) 6.19 (d, *J* = 2.5 Hz, H-6), 6.46 (d, *J* = 2.5 Hz, H-8), 6.85 (s, H-3), 7.5 (d, *J* = 2.5 Hz, H-2′), 6.89 (d, *J* = 8 Hz, H-5′), 7.45 (dd, *J* = 2.5 Hz and *J* = 8 Hz, H-6′). Comparison of the obtained spectral data of compound **5** with those reported for luteolin confirmed its identity [50]. Luteolin is reported here for the first time from *Erythrina crista-galli*.

### 2.1. Docking Study

**Figure 2:**
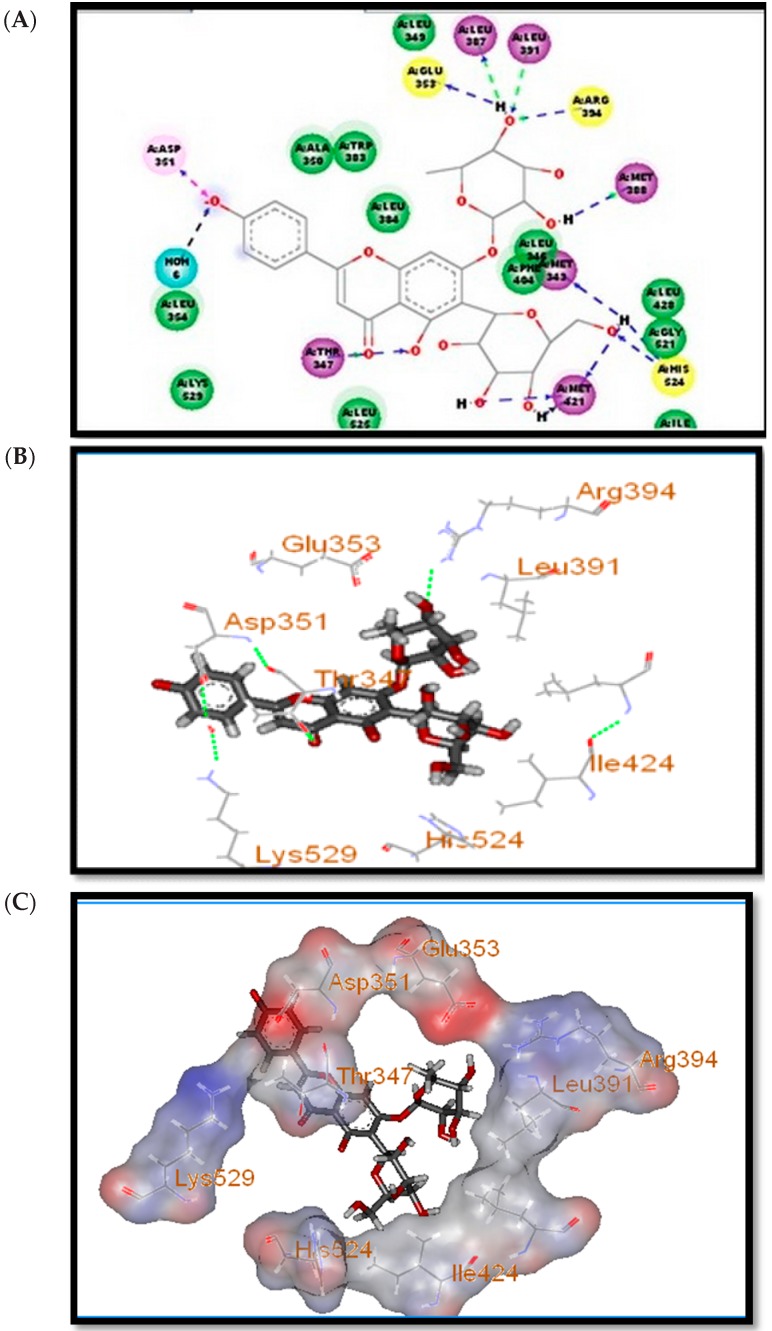
Binding mode for apigenin-7-*O*-rhamnosyl-6-*C*-glucoside on human estrogen receptor R. (**A**) 2D binding mode for apigenin-7-*O*-rhamnosyl-6-*C*-glucoside on human estrogen receptor R; (**B**) 3D binding mode for apigenin-7-*O*-rhamnosyl-6-*C*-glucoside on human estrogen receptor R; (**C**) apigenin-7-*O*-rhamnosyl-6-*C*-glucoside into the binding site surface of human estrogen receptor R.

The binding mode of compound E_2_ revealed formation of three hydrogen bonds with the residues Arg 394, Glu 353 and His 524 of the of human estrogen receptor R (ERR) (PDB ID 1A52) [51]. The isolated compounds were able to interact with the active site in the same pattern as the lead E_2_. The results showed that among the isolated compounds, apigenin-7-*O*-rhamnosyl-6-*C*-glucoside, apigenin-6-*C*-glucoside, luteolin-6-*C*-glucoside, apigenin and luteolin exhibit the same binding mode of the lead compound E_2_, with higher fitting scores (Table 1). The apigenin-7-*O*-rhamnosyl-6-*C*-glucoside binding mode is illustrated in (Figure 2A–C), with a fitting score of 44.7, which revealed an extra hydrogen bonding with more amino acids in the active pocket, in addition to the three key amino acids Arg 394, Glu 353 and His 524, indicating a firmer binding. However, the three compounds of interest share the same H-bonding of the lead compound with the same key amino acids, which suggests an identical binding mode with the active site with lead and, hence, probably the same biological activity. Interestingly, all the docked compounds are characterized by possessing one or more phenolic groups, which dissociate under physiological conditions resulting in O^−^ ions [1]. The polyphenols can form ionic bonds with positively charged side chains of the aspartate aminoacids (Asp 351).These bonds are quite weak, but because several of them are formed concomitantly, the effect is much stronger, leading to more fitting in the binding pocket. In addition, the docking study also revealed that simple phenyl-substituted coumarin derivatives are more likely to have the same binding mode as lead and hence the same biological activity. Accordingly, the molecular modeling studies that were performed indicated that the proposed molecules are promising candidates for phytoestrogenic activity, as compared to 17β-estradiol.

### 2.2. Phytoestrogenic Activity (pER8: GUS Reporter Assay)

The phytoestrogenic activity of the extract and the fractions as well as the reference compound 17β-estradiol were evaluated utilizing the *Arabidopsis thaliana* pER8: GUS reporter assay system [52]. The results of the minimal active concentration (MAC) of the 17β-estradiol (E_2_) and samples are presented in Figure 3. Fractions I and II exhibited the most obvious phytoestrogenic activity with MAC values lower than 6.25 µg/mL. Aqueous and aqueous methanol extracts showed less activity with MAC values of 25 and 12.5 µg/mL, respectively. The observed phytoestrogenic activity of *Erythrina crista-galli* and its fractions is supported by several *in vitro* and *in vivo* studies reporting the estrogenic activity of another *Erythrina* sp., *E. poeppigiana* [19,42]. In *E. lysistemon*, the phytoestrogenic activity is related to the prenylated flavonoid abyssinone *V*-4′-methyl-ether [53]. This finding is in agreement with our previous work showing the binding effect of prenylated flavonoids to the uterine estrogen receptor of rats [54]; the presence of a 5-hydroxy group and an 8-prenyl group were essential for such binding. In our previous papers, the reliability of the method was validated through comparing the phytoestrogenic results obtained by the pER8: GUS reporter assay with the results obtained by the standard *in vitro* model of the SEAP reporter assay system in the MCF-7 breast cancer cell-line [55,56]. The results of the pER8: GUS reporter assay were in agreement with the results of the SEAP reporter assay system in the MCF-7 breast cancer cell-line. However, the major limitation for this assay is its relative lower sensitivity (minimum active concentration of E_2_ at 1.25–0.63 nM) compared to the *in vitro* SEAP reporter assay (minimum active concentration 0.01 nM) [55].

### 2.3. Proliferation-Enhancing Activity in MCF-7 Cells

The phytoestrogenic activity of *Erythrina crista-galli* aqueous and aqueous methanol extracts and its active fractions (I and II) was further substantiated through assessing their effect on the proliferation of the estrogen-responsive cell line MCF-7. Initially, the cytotoxicity of the extracts and its active fractions (I and II) was determined using the sulphorhodamine B (SRB) assay. The aqueous extract showed an IC_50_ value of 187 ± 11.2 µg/mL, while that of the aqueous methanol extract was above 1000 µg/mL. IC_50_ values of fractions I and II were 23.3 ± 1.9 and 51.5 ± 3.5 µg/mL, respectively (Table 2). The aqueous extract of *Erythrina* was previously reported to possess cytotoxic activity against breast and lung cancer cell lines [39]. The observed superior activity of the aqueous extract over the aqueous methanol extract is referred to the presence of many other secondary metabolites in the multi-component plant extract. The presence of low amount of erythrinan (with their unique skeleton of a tetracyclic spiroamine) and benzylisoquinoline alkaloids may be regarded as the main reason for the relatively higher cytotoxic activity. These alkaloids showed IC_50_ values higher than 25 µg/mL when they were assessed against the murine macrophages RAW264.7 [46]. However, more potent activity of the total alkaloids fraction was noticed against HepG-2, HEP-2, HCT116, MCF-7 and HFB4, with IC_50_ values in the range of 2.97‒21.1 µg/mL [56].

**Figure 4:**
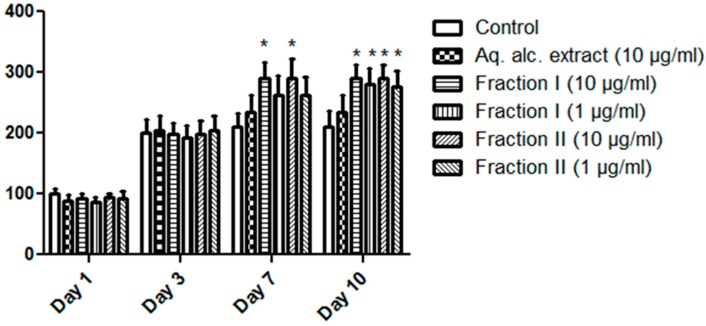
Effect of *Erythrina crista-galli* extract and fractions on the rate of proliferation of the MCF-7 cell line. * Significantly different from corresponding control at *p* < 0.05.

In this context, isolation of these alkaloids was not the scope of this work. The main objective was to evaluate the phytoestrogenic and cytoprotective activity of the plant extract. The aqueous methanol extract showed nontoxic effect on the tested cells, even in a high concentration. This can be attributed to the presence of only a low amount of the alkaloids for a solubility reason. However, the relatively higher cytotoxicity of the fractions may be attributed to the presence of other secondary metabolites, especially pterocarpan. These compounds showed potent inhibitory activity on protein-tyrosine phosphatase-1B, which is involved in many phosphorylation processes associated with cell growth and mitotic division [40]. Therefore, we selected the aqueous methanol extract together with fractions I and II for MCF-7 proliferation studies. The aqueous methanol extract (10 µg/mL) resulted in enhanced proliferation for MCF-7 cells. Sub-cytotoxic concentrations of fractions I and II (1 and 10 µg/mL) showed significant increases in the proliferation rate of MCF-7 cells at day 7 and day 10 (Table 3 and Figure 4). These results further support the phytoestrogenic activity evidenced by the *Arabidopsis thaliana* pER8: GUS reporter assay elaborated above.

## 3. Materials and Methods

### 3.1. Plant Material

Fresh leaves of *Erythrina crista-galli* L. were collected in April 2014 from plants grown in El-Giza Zoo garden, Giza. They were kindly authenticated morphologically by Mrs. Treese Labib, agricultural expert, El-Orman Botanical Garden, Giza, Egypt. Voucher specimens of the plant material were deposited at the Department of Pharmacognosy, Faculty of Pharmacy, Ain Shams University, Cairo, Egypt (P-NA-302).

### 3.2. Cell Lines and Chemicals

The human breast adenocarcinoma (MCF-7) cell line was obtained from the Egyptian Holding Company for Biological Products and Vaccines (VACSERA, Giza, Egypt). Ammonium nitrate, magnesium sulfate, potassium nitrate, boric acid, cobalt chloride, cupric sulfate, manganese sulfate, potassium iodine, sodium molybdate, zinc sulfate, citric acid and sodium citrate were purchased from Wako Pure Chemical Industries^®^ (Tokyo, Japan). Calcium chloride, dextrose, potassium hexacyanoferrate II, potassium hexacyanoferrate III and sodium carbonate were obtained from Merck^®^ (Darmstadt, Germany), while potassium phosphate, sodium chloride, ferrous sulfate, Na_2_-EDTA2H_2_O, nicotinic acid, pyridoxineHCl, thiamineHCl, Fe-EDTA, 4-morpholineethanesulfonic acid, τ-inositol, sucrose, phytoagar, sodium phosphate, silymarin, triton X-100, 5-bromo-4-chloro-3-indoly-β-d-glucuronide and α-Rhamnase were purchased from Sigma-Aldrich Corp. (St. Louis, MO, USA). 17β-estradiol was purchased from Takeda Chemical Industries Ltd. (Osaka, Japan). Solvents used were of analytical grade unless mentioned and were purchased from Merck, J.T. Backer^®^ (Deventer, The Netherlands) and Theo Seulberger^®^ (Karlsruhe, Germany). Media and supplements for cell cultures were obtained from Gibco^®^/Invitrogen (Karlsruhe, Germany) and Greiner Labortechnik^®^ (Frickenhausen, Germany).

### 3.3. Phytochemistry

^1^H- and ^13^C-NMR were measured on a Varian AC 500 MHz NMR spectrometer (Varian^®^, Santa Clara, CA, USA) at the operating frequencies of 500.13 and 125.67 MHz, respectively. Samples were dissolved in dimethyl sulphoxide (Deutero^®^, Kastellaun, Germany). The chemical shift (δ) values are reported in ppm in DMSO, and coupling constants (*J*-value) are recorded in Hz. UV recording was done on a Shimadzu UV Visible-1601 spectrophotometer, Shimadzu^®^ (Kyoto, Japan). The molecular weights were recorded using a Bruker micrOTOF-QII (Bruker Daltonics, Bremen, Germany) mass spectrometer operating at 70 eV with an ion source temperature 200 °C over the range 200–900 *m*/*z*. Chromatograms were processed using Bruker Compass Data Analysis 4.0 software.

Column chromatography (CC) was carried out on either silica gel 60 (0.063–0.2 mm) (Merck^®^, Darmstadt, Germany), polyamide S6, (Riedel-De Haen^®^, Hannover, Germany) and a Sephadex LH-20 (25–100 μm) (GE Healthcare Bio-Sciences^®^, Uppsala, Sweden). Fractions were monitored by thin layer chromatography (TLC) on either pre-coated silica gel 60 F254 (0.25 mm) (Merck^®^) using *p*-anisaldehyde/sulphuric acid reagent and heating at 100 °C for 7 min [57]. Paper chromatography (PC) was carried out on Whatmann No. 1 and No. 3 papers (Whatmann™, Kent, UK); localization of the spots on PC was performed by exposure to ammonia vapour.

### 3.4. Extraction and Isolation

*E. crista-galli* fresh leaves (1 kg) were chopped in double-distilled water (4 L) for 6 h three times. The aqueous extract was concentrated under reduced pressure followed by lyophilization. The dry lyophilized extract was then further extracted with methanol (HPLC grade) for 30 min at 40 °C to ensure complete extraction of the phenolic components. The extract was completely evaporated *in vacuo* (45 °C) until constant weight, to yield a solid residue (30 g).

Fractionation of the extract on a cellulose column using water, followed by water–methanol mixtures of decreasing polarities yielded two main fractions (I–II), which were individually subjected to further purification using sub-columns and preparative paper chromatography (PPC). Re-fractionation of fraction I over a polyamide 6S column and elution with H_2_O, followed by H_2_O/MeOH mixtures of decreasing polarities, yielded 6 main sub-fractions (I-A-IF). Compound **1** was obtained from sub-fraction I-C (eluted with 40% methanol) through subsequent fractionation over column chromatography using Sephadex LH-20 with H_2_O, followed by H_2_O/MeOH mixtures of decreasing polarities as the solvent system. Further purification was done using PPC eluted with *n*-butanol:acetic acid:water (BAW). Compounds **2** and **3** were obtained from sub-fractions I-D (eluted from 60% MeOH) through subsequent fractionation over a Sephadex LH-20 column, with *n*-butanol saturated with water as the solvent system. Further purification was done using PPC with BAW as the solvent system separating **2** from **3**. Compounds **4** and **5** were obtained from fraction II (eluted with neat MeOH) and purified through subsequent PPC using BAW as the solvent system [58].

### 3.5. Molecular Docking Study

The molecular docking study of the isolated compounds from the plant on the human estrogen receptor was carried out using Discovery Studio 2.5 software (Accelrys Inc., San Diego, CA, USA) Gold protocol. The X-ray crystal structure of human estrogen receptor R (ERR) (PDB ID 1A52) was downloaded from the Protein Data Bank (www.pdb.org). The structure of the receptor was established using the default protein preparation protocol of Accelry’s discovery studio 2.5 [59]. The study was started by determining the binding mode of bioactive conformation of the reported lead compound E_2_ crystallized with ER. The structure was obtained from the Protein Data Bank without change in its conformation to investigate the detailed intermolecular interactions between the ligand and the target receptor. A validation for the ideal pose was also performed by alignment of the X-ray bioactive conformer, with the best fitted pose of the same compound. The alignment showed good coincidence between them (RMSD = 0.21 Å), indicating the validity of the selected pose. Interactive docking using Gold protocol was carried out between the test set of molecules and the binding site of the prepared ER. Each test set molecule gave 10 possible docked poses. The ideal pose of each molecule was selected according to the similarity of its binding mode in the binding site to that of the lead compound. The binding pattern of the ideal pose for each of the test set molecules and the corresponding Gold fitness score were considered in our study to prioritize their virtual affinity to the binding site, in comparison to the ideal pose of the lead molecule E_2_. The predicted binding energies of the compounds are listed in Table 1.

### 3.6. Biological Activity

#### 3.6.1. Estrogenic Activity: pER8: GUS Reporter System

The *Arabidopsis* pER8: GUS reporter assay system was originally developed by Brand *et al.* [60]. The seeds of *Arabidopsis* pER8: GUS were sown and grown on MS solid medium (3% sucrose, 0.9% agar) in the dark for 24–36 h at 4 °C for vernalization, and then left at 24 °C for three days under continuous light. The seedlings were transferred into 24-well microtiter plates containing MS liquid medium with or without the tested compounds in each well, and incubated at 24 °C for 48 h. The culture medium was removed, then the staining buffer (50 mM Na_3_PO_4_ buffer (pH 7.0), 10 mM EDTA (pH 8.0), 0.2 mM X-Gluc, 0.5 mM K_3_Fe(CN)_6_, 0.5 mM K_4_Fe(CN)_6_ and 0.1% Triton X-100) was added for GUS staining at 37 °C for three hours [61]. The reference compound, 17β-estradiol, was used as a positive control (0.31–10 nM). A ZEISS Axiovert 200 inverted microscope (Carl Zeiss, Oberkochen, Germany) was used to examine GUS staining and images were captured with a digital camera.

#### 3.6.2. Cell Culture, Cytotoxicity and Cell Proliferation Assay

MCF-7 cells were maintained in Dulbecco’s minimal essential medium (DMEM), free of serum and phenol red and supplemented with 10% charcoal-stripped fetal bovine serum and 1% antibiotic-antimycotic solution. Cells were grown at 37 °C in a humidified atmosphere of 5% CO_2_. All experiments were performed with cells in the logarithmic growth phase.

Sensitivity to drugs was determined in triplicate using the SRB (sulforhodamine B) cell viability assay [62]. Exponentially growing MCF-7 cells were collected using 0.25% Trypsin-EDTA and plated in 96-well plates from Greiner Labortechnik^®^ at 1000 and 2000 cells/well, respectively. The cells were cultured for 24 h, then incubated with various concentrations (0.1–1000 µg/mL) of the tested samples (stock solution 1 mg/mL) at 37 °C for 72 h, and subsequently fixed with 10% trichloroacetic acid (TCA) for 1 h at 4 °C. After several washing steps, cells were exposed to 0.4% SRB for 10 min in the dark and subsequently washed with 1% glacial acetic acid. After drying overnight, Tris-HCl was used to dissolve the SRB-stained cells for 5 min on a shaker at 1600 rpm and optical density intensity was measured photometrically at 545 nm, using a microplate reader, ChroMate^®^ 4300, (Awareness Technology Inc., Palm City, FL, USA). The cell viability (%) of the three independent experiments was calculated using the Emax model as follows:

% Cell viability=(100−R)×(1−[D]mKdm+[D]m)+R

where R is the residual unaffected fraction (the resistance fraction), [D] is the drug concentration used, K_d_ is the drug concentration that produces a 50% reduction of the maximum inhibition rate and m is a Hill-type coefficient [47]. The assay was repeated with minor modification in order to evaluate the effect of sub-lethal concentrations (1 and 10 µg/mL) of fractions I and II and the aqueous methanol extract for up to 10 days. Cells were fixed at 1, 3, 7 and 10 days after exposure to different treatments.

#### 3.6.3. Statistical Analysis

All experiments were carried out three times unless mentioned otherwise. Continuous variables were presented as mean ± S.E. The IC_50_ was determined as the concentration of a sample, which resulted in a 50% reduction in cell viability or inhibition of the biological activity. IC_50_ values were calculated using a four-parameter logistic curve (SigmaPlot 11.0, Systat Software, Inc., Richmond, CA, USA), and all the data were sketched and statistically evaluated using one-way ANOVA followed by Tukey–Kramer multiple comparisons tests (GraphPad Prism 6.01, GraphPad Software, Inc., San Diego, CA, USA) when the significance value is <0.05 using the same significance level. The criterion for statistical significance was taken as *p* < 0.05.

## 4. Conclusions

This phytochemical study indicates the isolation of apigenin-7-*O*-rhamnosyl-6-*C*-glucoside as a new flavonoid glycoside and the isolation of luteolin-6-*C*-glucoside for the first time from *Erythrina crista-galli*. The plant exhibits interesting phytoestrogenic activities. Epidemiologal evidence indicates that the incidence of breast cancer is lower in Asian populations who consume high dietary concentrations of soy products which have a high phytoestogens content [63,64]. The current data give support to the traditional use of the herb in some ailments and warrant further investigations.

## Figures and Tables

**Table:** 

Compound	R_1_	R_2_	R_3_
Apigenin-7-*O*-rhamnosyl-6-*C*-glucoside (**1**)	H	glucose	rhamnose
Apigenin-6-*C*-glucoside (isovitexin) (**2**)	H	glucose	H
Luteolin-6-*C*-glucoside (Isoorientin) (**3**)	OH	glucose	H
Apigenin (**4**)	H	H	H
Luteolin (**5**)	OH	H	H

**Figure 3:** Phytoestrogenic effects of (**a**) 17β-estradiol (E_2_) and (**b**) Fractions I and II from *Erythrina crista-galli* in the *Arabidopsis thaliana* pER: GUS system. (**a**) Estrogenic activity of the reference compound 17β-estradiol was evaluated utilizing the pER8: GUS reporter assay system. MAC of 17β-estradiol was 2.5 nM; (**b**) Fractions I and II exhibited the most obvious estrogenic activity with the MAC values lower than 6.25 µg/mL. Aqueous and aqueous methanol extracts showed less activity with the MAC values of 25 and 12.5 µg/mL, respectively.

**Table:** 

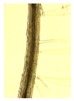	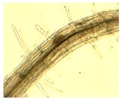	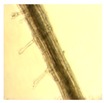	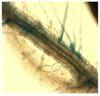	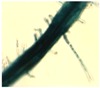	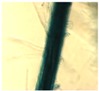	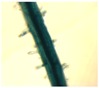
Blank	0.3125 nM	0.625 nM	1.25 nM	2.5 nM	5 nM	10 nM
(**a**)						
	6.25 µg/mL	12.5 µg/mL	25 µg/mL	50 µg/mL	100 µg/mL	200 µg/mL
**Aqueous extract**	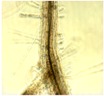	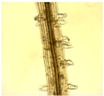	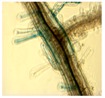	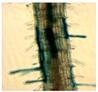	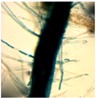	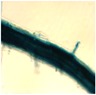
**Aqueous methanol extract**	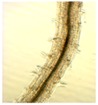	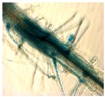	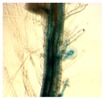	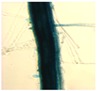	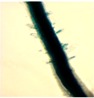	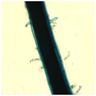
**Fraction I**	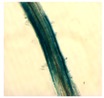	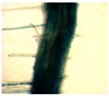	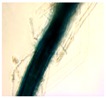	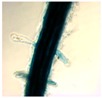	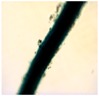	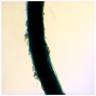
**Fraction II**	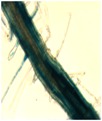	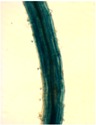	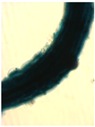	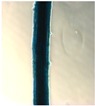	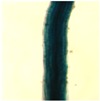	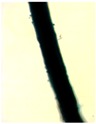
(**b**)						

**Table 1:** The computed binding energy values (Δ*G*_binding_) for the molecular docking study for the binding of the isolated components in *Erythrina crista-galli* extract with human Estrogen Receptor R (ERR).

Compound Name	Binding Energy (Δ*G*_binding_)
Apigenin-7-*O*-rhamnosyl-6-*C*-glucoside	−44.7
Apigenin-6-*C*-glucoside	−39.11
Luteolin-6-*C*-glucoside	−41.91
Apigenin	−43.33
Luteolin	−40.97
17β-estradiol	−36.38

The ligand-enzyme interaction energy value (∆*G*_binding_) was calculated using the following equation: ∆*G*_binding_ = *E*_complex_ − (*E*_ERRα_ + *E*_ligand_), where *E*_complex_ was the potential energy for the complex of human Estrogen Receptor R (ERR) bound with the ligand, *E*_ERRα_ was the potential energy of the receptor alone, and *E*_ligand_ was the potential energy for the ligand alone.

**Table 2:** IC_50_ values of extracts and fractions from *Erythrina crista-galli* on the growth of MCF-7 cells.

Addition	IC_50_ (µg/mL)
Aqueous extract	187.0 ± 11.2
Aqueous methanol extract	>1000
Fraction I	23.3 ± 1.9
Fraction II	51.5 ± 3.5

**Table 3:** The proliferation-enhancing activity of *Erythrina crista-galli* in MCF-7 cells using the SRB assay relative to the control.

Drug	Viability %			
	Day 1	Day 3	Day 7	Day 10
Control	100.00 ± 9.2	200.00± 22.92	210.97 ± 22.36	210.97 ± 25.36
Aq.alc. extract (10 µg/mL)	88.37 ± 9.63	204.87 ± 24.14	234.14 ± 29.02	234.14 ± 29.02
Fraction I (10 µg/mL)	91.46 ± 8.41	198.78 ± 18.7	290.24 ± 27.22 *	290.24 ± 21.22 *
(1 µg/mL)	86.58 ± 7.19	193.90 ± 20.26	261.95 ± 32.12	279.83 ± 26.34 *
Fraction II (10 µg/mL)	93.41 ± 5.97	198.78 ± 21.66	290.24 ± 31.22 *	290.24 ± 21.22 *
(1 µg/mL)	92.68 ± 10.85	203.66 ± 24.14	262.73 ± 30.12	276.83 ± 26.34 *

Data are represented in terms of mean ± SEM (*n* = 3). * Significantly different from control at *p* < 0.05.

## References

[1] Wink M. (2008). Evolutionary advantage and molecular modes of action of multi-component mixtures used in phytomedicine. Curr. Drug Metab..

[2] Psotova J., Kolář M., Soušek J., Švagera Z., Vičar J., Ulrichová J. (2003). Biological activities of *Prunella vulgaris* extract. Phytother. Res..

[3] Fukumoto L., Mazza G. (2000). Assessing antioxidant and prooxidant activities of phenolic compounds. J. Agric. Food Chem..

[4] Nomura T., Fukai T., Akiyama T. (2002). Chemistry of phenolic compounds of licorice (*Glycyrrhiza* species) and their estrogenic and cytotoxic activities. Pure Appl. Chem..

[5] Miksicek R.J. (1993). Commonly occurring plant flavonoids have estrogenic activity. Mol. Pharmacol..

[6] Rao Y.K., Geethangili M., Fang S.-H., Tzeng Y.-M. (2007). Antioxidant and cytotoxic activities of naturally occurring phenolic and related compounds: A comparative study. Food Chem. Toxicol..

[7] Yoshikawa M., Ninomiya K., Shimoda H., Nishida N., Matsuda H. (2002). Hepatoprotective and antioxidative properties of *Salacia reticulata*: Preventive effects of phenolic constituents on CCl_4_-induced liver injury in mice. Biol. Pharm. Bull..

[8] Jain A., Soni M., Deb L., Jain A., Rout S., Gupta V., Krishna K. (2008). Antioxidant and hepatoprotective activity of ethanolic and aqueous extracts of *Momordica dioica* Roxb. Leaves. J. Ethnopharmacol..

[9] Hegnauer R., Renpe J., Gpayer-Barkmeijer R.J. (1993). Relevance of seed polysaccharides and flavonoids for the classification of the leguminosae: A chemotaxonomic approach. Phytochemistry.

[10] Nestel P.J., Pomeroy S., Kay S., Komesaroff P., Behrsing J., Cameron J.D., West L. (1999). Isoflavones from red clover improve systemic arterial compliance but not plasma lipids in menopausal women. J. Clin. Endocrinol. Metab..

[11] Tindale M.D., Roux D. (1969). A phytochemical survey of the Australian species of *Acacia*. Phytochemistry.

[12] Li W., Asada Y., Yoshikawa T. (2000). Flavonoid constituents from *Glycyrrhiza glabra* hairy root cultures. Phytochemistry.

[13] Wang G.-S., Han Z.-W. (1993). Effects of flavonoids of *glycyrrhiza* on ethanol-induced liver injury in mice. Chin. Pharmacol. Bull..

[14] Kone W.M., Solange K.-N.E., Dosso M. (2011). Assessing sub-Saharan *Erythrina* for efficacy: Traditional uses, biological activities and phytochemistry. Pak. J. Biol. Sci..

[15] De Lima M.R.F., de Souza Luna J., dos Santos A.F., de Andrade M.C.C., Sant’Ana A.E.G., Genet J.-P., Marquez B., Neuville L., Moreau N. (2006). Anti-bacterial activity of some Brazilian medicinal plants. J. Ethnopharmacol..

[16] Flausino O.A., Pereira A.M., da Silva Bolzani V., Nunes-de-Souza R.L. (2007). Effects of erythrinian alkaloids isolated from *Erythrina mulungu* (Papilionaceae) in mice submitted to animal models of anxiety. Biol. Pharm. Bull..

[17] Juma B.F., Majinda R.R.T. (2004). Erythrinaline alkaloids from the flowers and pods of *Erythrina lysistemon* and their DPPH radical scavenging properties. Phytochemistry.

[18] Wanjala C.C., Juma B.F., Bojase G., Gashe B.A., Majinda R.R. (2002). Erythrinaline alkaloids and antimicrobial flavonoids from *Erythrina latissima*. Planta Med..

[19] Djiogue S., Halabalaki M., Njamen D., Kretzschmar G., Lambrinidis G., Hoepping J., Raffaelli F.M., Mikros E., Skaltsounis A.L., Vollmer G. (2014). Erythroidine alkaloids: A novel class of phytoestrogens. Planta Med..

[20] Hikita K., Tanaka H., Murata T., Kato K., Hirata M., Sakai T., Kaneda N. (2014). Phenolic constituents from stem bark of *Erythrina poeppigiana* and their inhibitory activity on human glyoxalase I. J. Nat. Med..

[21] KamdemWaffo A.F., Coombes P.H., Mulholland D.A., Nkengfack A.E., Fomum Z.T. (2006). Flavones and isoflavones from the west African Fabaceae *Erythrina vogelii*. Phytochemistry.

[22] Tanaka H., Atsumi I., Shirota O., Sekita S., Sakai E., Sato M., Murata J., Murata H., Darnaedi D., Chen I.S. (2011). Three new constituents from the roots of *Erythrina variegata* and their antibacterial activity against methicillin-resistant *Staphylococcus aureus*. Chem. Biodivers..

[23] Chukwujekwu J.C., van Heerden F.R., van Staden J. (2011). Antibacterial activity of flavonoids from the stem bark of *Erythrina caffrathunb*. Phytother. Res..

[24] Innok P., Rukachaisirikul T., Phongpaichit S., Suksamrarn A. (2010). Fuscacarpans A–C, new pterocarpans from the stems of *Erythrina fusca*. Fitoterapia.

[25] Dao T.T., Nguyen P.H., Thuong P.T., Kang K.W., Na M.K., Ndinteh D.T., Mbafor J.T., Oh W.K. (2009). Pterocarpans with inhibitory effects on protein tyrosine phosphatase 1B from *Erythrina lysistemon* Hutch. Phytochemistry.

[26] Innok P., Rukachaisirikul T., Suksamrarn A. (2009). Flavanoids and pterocarpans from the bark of Erythrinafusca. Chem. Pharm. Bull..

[27] Watjen W., Suckow-Schnitker A.K., Rohrig R., Kulawik A., Addae-Kyereme J., Wright C.W., Passreiter C.M. (2008). Prenylated flavonoid derivatives from the bark of *Erythrina addisoniae*. J. Nat. Prod..

[28] Jang J., Na M., Thuong P.T., Njamen D., Mbafor J.T., Fomum Z.T., Woo E.R., Oh W.K. (2008). Prenylated flavonoids with PTP1B inhibitory activity from the root bark of *Erythrina mildbraedii*. Chem. Pharm. Bull..

[29] Cui L., Thuong P.T., Lee H.S., Njamen D., Mbafor J.T., Fomum Z.T., Lee J., Kim Y.H., Oh W.K. (2008). Four new chalcones from *Erythrina abyssinica*. Planta Med..

[30] Na M., Jang J., Njamen D., Mbafor J.T., Fomum Z.T., Kim B.Y., Oh W.K., Ahn J.S. (2006). Protein tyrosine phosphatase-1B inhibitory activity of isoprenylated flavonoids isolated from *Erythrina mildbraedii*. J. Nat. Prod..

[31] Sato M., Tanaka H., Yamaguchi R., Oh-Uchi T., Etoh H. (2003). Erythrinapoeppigiana-derived phytochemical exhibiting antimicrobial activity against *Candida albicans* and methicillin-resistant *Staphylococcus aureus*. Lett. Appl. Microbiol..

[32] Iinuma M., Okawa Y., Tanaka T. (1994). Three new cinnamylphenols in heartwood of *Erythrina crista-galli*. Phytochemistry.

[33] Tanaka H., Sudo M., Kawamura T., Sato M., Yamaguchi R., Fukai T., Sakai E., Tanaka N. (2010). Antibacterial constituents from the roots of *Erythrina herbacea* against methicillin-resistant *Staphylococcus aureus*. Planta Med..

[34] Andayi A.W., Yenesew A., Derese S., Midiwo J.O., Gitu P.M., Jondiko O.J.I., Akala H., Liyala P., Wangui J., Waters N.C. (2006). Antiplasmodial flavonoids from *Erythrina sacleuxii*. Planta Med..

[35] Sakat S., Juvekar A. (2010). Comparative study of *Erythrina indica* Lam. (Febaceae) leaves extracts for antioxidant activity. J. Young Pharm..

[36] Njamen D., Mbafor J.T., Fomum Z.T., Kamanyi A., Mbanya J.C., Recio M.C., Giner R.M., Manez S., Rios J.L. (2004). Anti-inflammatory activities of two flavanones, sigmoidin A and sigmoidin B, from *Erythrina sigmoidea*. Planta Med..

[37] Agrawal S.K., Agrawal M., Sharma P.R., Gupta B.D., Arora S., Saxena A.K. (2011). Induction of apoptosis in human promyelocytic leukemia HL60 cells by an extract from *Erythrina suberosa* stem bark. Nutr. Cancer.

[38] Kuete V., Sandjo L.P., Djeussi D.E., Zeino M., Kwamou G.M., Ngadjui B., Efferth T. (2014). Cytotoxic flavonoids and isoflavonoids from *Erythrina sigmoidea* towards multi-factorial drug resistant cancer cells. Investig. New Drugs.

[39] RathiSre P.R., Reka M., Poovazhagi R., Arul Kumar M., Murugesan K. (2015). Antibacterial and cytotoxic effect of biologically synthesized silver nanoparticles using aqueous root extract of *Erythrina indica* Lam. Spectrochim. Acta A Mol. Biomol. Spectrosc..

[40] Nguyen P.H., Le T.V., Thuong P.T., Dao T.T., Ndinteh D.T., Mbafor J.T., Kang K.W., Oh W.K. (2009). Cytotoxic and PTP1B inhibitory activities from *Erythrina abyssinica*. Bioorg. Med. Chem. Lett..

[41] Djiogue S., Njamen D., Halabalaki M., Kretzschmar G., Beyer A., Mbanya J.C., Skaltsounis A.L., Vollmer G. (2010). Estrogenic properties of naturally occurring prenylated isoflavones in U2OS human osteosarcoma cells: Structure-activity relationships. J. Steroid. Biochem. Mol. Biol..

[42] Njamen D., Djiogue S., Zingue S., Mvondo M.A., Nkeh-Chungag B. (2013). *In vivo* and *in vitro* estrogenic activity of extracts from *Erythrina poeppigiana* (Fabaceae). J. Complement. Integr. Med..

[43] Zhang Y., Li Q., Li X., Wan H.Y., Wong M.S. (2010). *Erythrina variegata* extract exerts osteoprotective effects by suppression of the process of bone resorption. Br. J. Nutr..

[44] Vasconcelos S.M., Macedo D.S., de Melo C.T., Paiva Monteiro A., Rodrigues A.C., Silveira E.R., Cunha G.M., Sousa F.C., Viana G.S. (2004). Central activity of hydro alcoholic extracts from *Erythrina velutina* and *Erythrin amulungu* in mice. J. Pharm. Pharmacol..

[45] Hashimoto G. (1996). Illustrated Cyclopedia of Brazilian Medicinal Plants.

[46] Ozawa M., Kawamata S., Etoh T., Hayashi M., Komiyama K., Kishida A., Kuroda C., Ohsaki A. (2010). Structures of new erythrinan alkaloids and nitric oxide production inhibitors from *Erythrina crista-galli*. Chem. Pharm. Bull..

[47] Ramarathnam N., Osawa T., Namiki M., Kawakishi S. (1989). Chemical studies on novel rice hull antioxidants. 2. Identification of isovitexin, a *C*-glycosyl flavonoid. J. Agric. Food Chem..

[48] Koeppen B., Roux D. (1965). *C*-Glycosylflavonoids. The chemistry of orientin and iso-orientin. Biochem. J..

[49] Peng J., Fan G., Hong Z., Chai Y., Wu Y. (2005). Preparative separation of isovitexin and isoorientin from *Patrinia villosa* Juss by high-speed counter-current chromatography. J. Chromatogr. A.

[50] Mabry T.J., Markham K.R., Thomas M.B. (1970). The Systematic Identification of Flavonoids.

[51] Celik L., Lund J.D., Schiøtt B. (2007). Conformational dynamics of the estrogen receptor α: Molecular dynamics simulations of the influence of binding site structure on protein dynamics. Biochem. J..

[52] Chang F.R., Hayashi K., Chua N.H., Kamio S., Huang Z.Y., Nozaki H., Wu Y.C. (2005). The transgenic *Arabidopsis* plant system, pER8-GFP, as a powerful tool in searching for natural product estrogen-agonists/antagonists. J. Nat. Prod..

[53] MagneNde C.B., Njamen D., TaneeFomum S., Wandji J., Simpson E., Clyne C., Vollmer G. (2012). *In vitro* estrogenic activity of two major compounds from the stem bark of *Erythrina lysistemon* (Fabaceae). Eur. J. Pharmacol..

[54] Hillerns P.I., Wink M. (2005). Binding of flavonoids from *Sophora flavescens* to the rat uterine estrogen receptor. Planta Med..

[55] Tsai Y.C., Chiang S.Y., El-Shazly M., Wu C.C., Beerhues L., Lai W.C., Wu S.F., Yen M.H., Wu Y.C., Chang F.R. (2013). The oestrogenic and anti-platelet activities of dihydrobenzofuroisocoumarins and homoisoflavonoids from *Liriope platyphylla* roots. Food Chem..

[56] Mohammed M.M., Ibrahim N.A., Awad N.E., Matloub A.A., Mohamed-Ali A.G., Barakat E.E., Mohamed A.E., Colla P.L. (2012). Anti-HIV-1 and cytotoxicity of the alkaloids of *Erythrina abyssinica* Lam. growing in Sudan. Nat. Prod. Res..

[57] Wagner H., Bladt S. (2009). Plant Drug Analysis, A Thin Layer Chromatography Atlas.

[58] Hassaan Y., Handoussa H., El-Khatib A.H., Linscheid M.W., El Sayed N., Ayoub N. (2014). Evaluation of plant phenolic metabolites as a source of Alzheimer’s drug leads. Biomed. Res. Int..

[59] Tanenbaum D.M., Wang Y., Williams S.P., Sigler P.B. (1998). Crystallographic comparison of the estrogen and progesterone receptor’s ligand binding domains. Proc. Natl. Acad. Sci. USA.

[60] Brand L., Hörler M., Nüesch E., Vassalli S., Barrell P., Yang W., Jefferson R.A., Grossniklaus U., Curtis M.D. (2006). A versatile and reliable two-component system for tissue-specific gene induction in *Arabidopsis*. Plant Physiol..

[61] Lai W.C., Wang H.C., Chen G.Y., Yang J.C., Korinek M., Hsieh C.J., Nozaki H., Hayashi K.I., Wu C.C., Wu Y.C. (2011). Using the pER8: GUS reporter system to screen for phytoestrogens from *Caesalpinia sappan*. J. Nat. Prod..

[62] Skehan P., Storeng R., Scudiero D., Monks A., McMahon J., Vistica D., Warren J.T., Bokesch H., Kenney S., Boyd M.R. (1990). New colorimetric cytotoxicity assay for anticancer-drug screening. J. Natl. Cancer Inst..

[63] Mense S.M., Hei T.K., Ganju R.K., Bhat H.K. (2008). Phytoestrogens and breast cancer prevention: Possible mechanisms of action. Environ. Health Perspect..

[64] Miller P.E., Snyder D.C. (2012). Phytochemicals and cancer risk: A review of the epidemiological evidence. Nutr. Clin. Pract..

